# Community leaders’ experiences of hepatitis E in a Namibian informal settlement: A qualitative study

**DOI:** 10.4102/jphia.v16i1.1359

**Published:** 2025-07-09

**Authors:** Abraham V. Nghikevali, Talitha Crowley

**Affiliations:** 1 School of Nursing, Faculty of Community and Health Science, University of the Western Cape, Bellville, South Africa

**Keywords:** community, community leaders, experiences, Havana informal settlement, hepatitis E, spread

## Abstract

**Background:**

The hepatitis E virus (HEV) is a major global health concern, with an estimated one-third of the human population infected. It is particularly prevalent in developing countries, especially in informal settlements where inadequate sanitation and limited access to clean water contribute to its spread.

**Aim:**

The study aimed to explore and describe community leaders’ experiences regarding the spread of HEV in the Havana informal settlement, Khomas region, Namibia.

**Setting:**

This study focused on Havana informal settlement in the Namibian capital of Windhoek, which is in the Khomas region. The Havana informal settlement is predominantly populated with unemployed people.

**Methods:**

Individual in-depth interviews were conducted with 15 community leaders, aged 24 years to 65 years, who were knowledgeable of the spread of HEV in the informal settlement. Thematic qualitative analysis was used to analyse data.

**Results:**

The themes identified included HEV risk awareness, contextual factors influencing the spread of HEV, action drivers for change and actions taken to activate change. Overall, there was a good understanding of HEV, although some individuals in the community held incorrect information and harboured myths and misconceptions about HEV. Inadequate access to water, sanitation facilities and unhygienic cultural practices were identified as primary routes of transmission, fuelled by overcrowding.

**Conclusion:**

The study highlighted the importance of government-private sector cooperation, community health education, access to clean water and improved infrastructure in preventing the spread of HEV. Addressing these factors is essential for overcoming health challenges in informal settlements.

**Contribution:**

This research underscores the critical role of community engagement in mitigating HEV outbreaks in informal settlements.

## Introduction

Hepatitis E virus (HEV) refers specifically to the causative agent responsible for hepatitis E infection, while, hepatitis E is a viral infection that leads to liver inflammation and damage, primarily transmitted through the faecal-oral route.^1^

Characterised by an acute onset of jaundice, fever, malaise, nausea and anorexia, the incubation period for HEV ranges from 3 weeks to 8 weeks.^2^ The inflammation associated with the infection results in swelling of the liver, which can occur when tissues or organs are injured or infected. Hepatitis E virus is an emerging and re-emerging viral disease affecting both humans and various domestic and wild animals.^3^ There has been a notable increase in HEV epidemics in many developing countries, particularly because of factors such as rural-urban migration and poverty, which have not been effectively addressed.^4^ These socio-economic challenges have contributed to the proliferation of informal settlements in urban areas.^5^ Vulnerable populations, including pregnant women and individuals with compromised immune systems, are particularly at risk for infection.^6^ In Northern Uganda, HEV infection was most prevalent among individuals aged 15 years to 45 years, while those under 15 years exhibited asymptomatic cases.^6^ Alarmingly, pregnant women in their third trimester faced high fatality rates, with a reported 20% mortality among HEV-infected pregnant women in the region. The prognosis for HEV is generally favourable for individuals without comorbidities; however, pregnant women, children and those with underlying health conditions are at a higher risk for severe symptoms and related morbidity.^7^

Hepatitis E virus is a significant public health concern, particularly in resource-limited areas with inadequate access to clean water and sanitation. Genotypes 1 and 2, which are mainly spread by the faecal-oral route, are most frequently responsible for outbreaks of the HEV, which is a serious public health problem, especially in places with low resources and poor access to clean water and sanitation.

The virus is primarily transmitted through the faecal-oral route, often linked to contaminated drinking water sources.^8^ The World Health Organization (WHO) emphasises the importance of promoting food safety and environmental hygiene as effective preventive measures against HEV.^9^ Although a vaccine exists, it is not available in Namibia, where the latest outbreak occurred in December 2017 in the Havana informal settlement of Windhoek.^10^

Globally, HEV is recognised as an emerging cause of acute hepatitis, particularly in low-income regions, including parts of South America and Asia, where it is the second most significant cause of acute clinical hepatitis in adults.^11^ Hepatitis E virus was first identified in the 1980s during an outbreak among Soviet soldiers in Afghanistan.^12^ The virus is endemic in many regions of Africa and Asia,^13^ and in developed countries, cases are often associated with travel to endemic areas.^14^ In many regions, including Europe and the United States (US), HEV is often acquired through the consumption of undercooked meat products, highlighting the potential for zoonotic transmission.^15^ In developing countries like Namibia, HEV is prevalent in overcrowded areas with limited sanitation and clean water access.^16^ The virus spreads through the faeces of infected individuals, and inadequate sanitation facilities exacerbate transmission risks. Proper hygiene practices are crucial to prevent virus ingestion, particularly in informal settlements where socio-economic conditions are poor.^17^

The 2017 outbreak in Namibia resulted in 127 laboratory-confirmed cases and 2342 epidemic-linked cases, with 26 reported deaths.^10^ Beginning in December 2017, Namibia saw a serious hepatitis E outbreak that expanded to all 14 regions, resulting in over 8000 cases and 66 fatalities.^18^ On 02 March 2022, the outbreak was formally declared to be over. No reports of minor outbreaks have surfaced since. It is crucial to remember that hepatitis E can endure in places with poor sanitation and little access to potable water, which are circumstances that exist in certain Namibian communities.^19^ Thus, even if no new outbreaks have been documented, there is still a chance that instances could occur in the future, particularly in susceptible locations.

The Havana informal settlement, home to approximately 40 000 residents, faced significant challenges because of unemployment and inadequate sanitation.^20^ In response to the outbreak, the Namibian Ministry of Health implemented measures to reduce HEV infections, including providing sanitation facilities and public education.^10^ Community health workers were recruited to enhance health education, and a national health emergency committee was established to coordinate efforts.^10^ Community leaders played a vital role in identifying health threats and promoting healthier lifestyles, significantly contributing to the prevention of HEV transmission in the affected areas.

Hepatitis E virus remains a global health concern,^21^ and while it can be prevented through effective community actions, research on the topic remains limited. Hence this study aimed to explore the community leaders’ experiences regarding the spread of HEV in the Havana informal settlement, Khomas region, Namibia. Understanding their insights can strengthen community-led interventions and inform more effective public health strategies.

### Theoretical framework

The health belief model (HBM) helps explain health behaviours by examining individuals’ perceptions of risk and the benefits of taking preventive actions.^22^ It suggests people are more likely to act if they believe they are vulnerable to a health threat, perceive it as severe and see the action as effective. The model also considers barriers to action and self-efficacy, or confidence in one’s ability to take action. This framework was used in this study to provide insight into community leaders’ experiences of the spread of HEV in their communities.

## Research methods and design

### Design, study population and sampling techniques

A qualitative, exploratory, descriptive and contextual methodology was used. Community leaders were purposively selected to participate in the study. Eligibility requirements included being a section head overseeing one of Havana’s 26 sections for a minimum of 2 years to 3 years, acting as a health focal point since the Ministry of Health and Social Services (MOHSS) announced the HEV outbreak, being available at the time of the study and consenting to participate. With the assistance of the community leaders, 17 eligible participants were contacted of which 15 consented to participate in the study. The lead researcher built a working connection between some of the participants prior to community health engagement programmes in Havana. However, reflexivity was maintained throughout the study to reduce any bias in how participants’ replies were interpreted. This familiarity helped to build trust and encouraged open, honest dialogue during interviews. Data saturation occurred at the 13th interview, where no new themes emerged. However, interviews were continued with all 15 participants to ensure rich and comprehensive data coverage.

### Instruments and data gathering

Individual in-depth interviews were conducted by the first author using a semi-structured interview guide between September 2021 and October 2022. It was available in both Oshiwambo and English. A language expert conducted forward and backward translations of the interview guide, and the first author, who is fluent in both languages, reviewed it for accuracy and consistency. Although one participant spoke Kavango, he preferred to be interviewed in English, which he understood well; therefore, no additional translation of the guide into Kavango was necessary. Prior to the main study, the interview guide was pilot-tested with three community leaders from a neighbouring area not included in the final sample. This helped to assess the clarity, cultural appropriateness and relevance of the questions, leading to minor adjustments in wording and sequencing. Participants who participated in the pilot study were not included in the main study. The interviews lasted between 30 min and 60 min and were held at a private location decided upon by the participant and the interviewer.

### Trustworthiness

Trustworthiness, which emphasises credibility, transferability, dependability and confirmability, guarantees that qualitative research is impartial, trustworthy and dependable.^23^ In order to establish trustworthiness, the researcher, who has lived in the Havana community for a long time, had prolonged engagement with study participants for 1 year. Four participants participated in member checking, during which the findings were discussed with them. Credibility was increased by an audit trail, peer debriefing and author agreement on coding. Transferability was attained by using verbatim quotes from participants and contextual explanations to validate interpretations. Documenting processes and keeping an audit trail guaranteed dependability, while admitting study limits encouraged transparency. In order to reduce bias, personal assumptions were documented in a reflective journal, and confirmability was attained by reflexivity and bracketing.

### Data analysis

Deductive thematic analysis was employed using the HBM as a framework.^24^ The identification and coding of themes were driven by predefined HBM categories, which included perceived vulnerability, severity, benefits, barriers, cues to action and self-efficacy. An independent coder who was not one of the co-authors undertook an inquiry audit, and a consensus was reached on the themes and sub-themes. The independent coder was trained in qualitative analysis to enhance credibility and ensure consistency in theme identification. Any discrepancies in coding were discussed and resolved through consensus. In order to provide a theory-driven approach, data analysis concentrated on comprehending perceived HEV risk, the efficacy of preventive measures, and implementation problems. Predetermined HBM categories were used in the study to direct interview questions and data analysis. Each construct was evaluated using focused questions that probed participants’ perspectives and experiences regarding the HEV outbreak. To find trends in perceived risk, the efficacy of preventive measures, and implementation difficulties, transcripts were deductively classified based on these categories.

Two Oshiwambo original transcripts were translated into English, and their accuracy was confirmed. After analysing the verbatim transcriptions, important accounts were found and categorised into themes with related meanings. Four participants’ member checks validated the themes.

### Ethical considerations

The University of the Western Cape’s Biomedical Research Ethics Committee approved this project (ethical clearance number: BM21/4/10). Before the study started, permission to perform it was received from Namibia’s Ministry of Health and Social Services (reference: 17/3/AN, dated 13 August 2021). All participants signed an informed consent form before being included in the study, which guaranteed participant autonomy.

## Results

### Participant biographical data

Fifteen community leaders were interviewed with ages ranging between 24 years and 65 years. Of the 15 participants, the majority spoke Oshiwambo as their home language (six females and eight males), while one male spoke Kavango. When looking at employment status, the data showed that only three participants, all of whom were men, were employed. Participants’ educational backgrounds differed. Eleven participants, four men and seven women, had finished their basic education, compared to one who had no formal schooling. Furthermore, three participants, two female and one male, had completed postsecondary education. Five participants had lived in Havana settlement for 5 years to 10 years, while one person had stayed there for 2 years to 4 years. Nine participants, two males and seven females, made up the largest group and had been in the settlement for almost a decade.

### Themes and sub-themes

**FIGURE 1:**
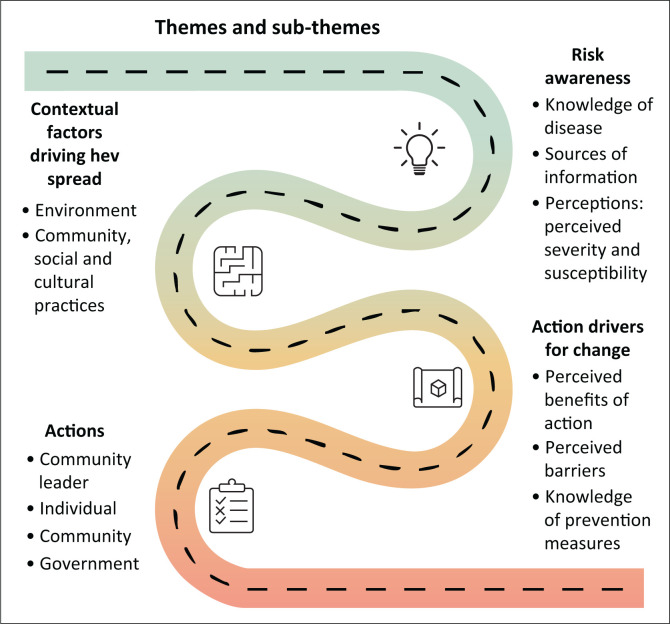
Major themes and sub-themes.

The study’s analysis revealed four main themes, each of which is illustrated with statements taken directly from the participants (Figure 1).

Participants were aware of the risk in the community, which prompted a variety of measures to stop the spread of HEV. Nonetheless, contextual elements and action drivers had an impact on these behaviours.

#### Theme 1: Risk awareness

The theme risk awareness, which had sub-themes such as disease knowledge, information sources, severity perceptions and perceived susceptibility, embodied participants’ comprehension of HEV and their risk of infection.

**Knowledge of the disease:** Knowledge of HEV is an important first step in the prevention of HEV at the community level. When community members are knowledgeable about the disease, they are likely to participate in their own health. Most participants in this study expressed their knowledge of the disease, which included the signs and symptoms:

‘Signs and symptoms of HEV are yellow eyes, abdominal pain and body weakness.’ (Participant 3, male, age 45 years)

The faecal-oral pathway of HEV infection was acknowledged by all participants, who gave examples of improper faecal disposal because of a shortage of restrooms, contaminated water sources and defecating close to riverbeds:

‘I am thinking it transmit as we defecate in the river during the night and morning, poor sanitation as we don’t have enough toilets and inadequate clean water source since we gather at one tap as a community to fetch water.’ (Participant 1, male, age 45 years)

Although most of the community leaders appeared to have adequate knowledge of HEV, they highlighted that there was a general lack of awareness and inadequate knowledge in the community:

‘Contributing factors to HEV spread I would say basically just lack of awareness, education to our community such as poor hygiene after use of toilet, poor food handling hygiene and lack of environmental hygiene.’ (Participant 10, male, age 52 years)

Community leaders also indicated that some community members were misinformed and needed more information about HEV:

‘Yes, at this point we need more effective information about the good sanitation practices and how to keep ourselves vigilant about these diseases and everything that causes it as however some people aware but they do not follow the HEV prevention protocols such as consistent uses of are toilet.’ (Participant 8, female, age 52 years)

Sources of information: Information sources, such as speeches, documents, photographs, organisations and observations, impart knowledge or educate people.^23^ In order to inform and prevent HEV transmission, community leaders mostly obtained information on HEVs from medical professionals, other community leaders and press outlets like local radio:

‘We heard information about HEV from the Ministry of Health and Social Services workers, from nearest health facilities, community health worker, local radio, and the information was on signs symptom, mode of transmission and prevention measures within community.’ (Participant 8, female, age 52 years)

**Perceptions: Perceived severity of the disease:** The term perceived severity describes the adverse effects of HEV, including possible long-term repercussions like the prognosis of the disease.^21^ Participants acknowledged that HEV may cause major problems for some people, including liver disorders:

‘In some people with HEV ended with prolonged liver problem and poor recovery.’ (Participant 11, male, age 41 years)

Participants noted that HEV infection could have long-term effects, such as chronic liver disease and liver failure, potentially leading to death. Some were aware of community members who had died from the disease:

‘HEV is dangerous disease. It can cause liver failure. We have community members who died due to HEV.’ (Participant 2, female, age 40 years)

The participants indicated they have experienced HEV-related deaths in their community, as well as long hospitalisation that had impacted families and individuals:

‘The HEV affected our community because of prolonged hospitalization, due to sickness and some died due to HEV.’ (Participant 11, male, age 41 years).

**Perceptions: Perceived susceptibility to disease:** Beliefs on the probability of contracting an illness are referred to as perceived susceptibility.^2^ Although community leaders acknowledged the population’s susceptibility to HEV, they pointed out that some people continued dangerous behaviours that enhanced their exposure as they did not comprehend the disease:

‘Despite community health education given to the informal settlement community members some people continued practising cultural unhygienic hand washing practices and use of liver bed for defecation and washing hands in one open bucket together.’ (Participant 10, female, age 40 years)

Participants acknowledged that everyone is susceptible to HEV but perceived pregnant women and children as more vulnerable and likely to experience worse outcomes. This perception stemmed from either their observations or information they had received:

‘It is mostly affecting the children and pregnant women. The disease infects these pregnant people, get sick faster, that is the same for children.’ (Participant 2, female, age 49 years)

Community leaders recognised that people in informal settlements are particularly susceptible to HEV because of socio-economic factors such as overcrowding, limited sanitation facilities and poor environmental hygiene:

‘In the informal settlement people at risk because overcrowding, with few toilets, poor hygiene so these are main factor that will also continue to increase the chances of getting HEV infections.’ (Participant 3, male, age 33 years)

Participants said that throughout the outbreak, families that were either directly or indirectly impacted by HEV became more conscious of their risk, which raised their awareness of their susceptibility and changed their behaviour:

‘At first, people took it very lightly and ignored the given facts about HEV, people were not even adhering to the hepatitis E prevention protocol such as washing their hands, use of toilet but later when the virus started infecting the community within their surroundings, they started following protocol such as regular hand washing.’ (Participant 1, male, age 45 years)

#### Theme 2: Contextual factors that drive hepatitis E virus spread

This theme focuses on participants’ awareness of contextual factors, including the study area’s geography and social determinants of health contributing to HEV spread. Sub-themes include environmental factors and community, social and cultural practices.

**Poor environmental hygiene within the community:** Poor hygiene and environmental health are major contributors to HEV spread. Participants highlighted that inadequate waste management and sanitation in informal communities have facilitated the spread of the disease:

‘Environmental health became a challenge because inadequate sanitation facilities, such as public toilets, use of riverbeds for defecation, lack of dustbin or formal dumping in the Havana informal settlement.’ (Participant 14, male, age 41 years)

Poor sanitation in the informal settlement is one of the major contributing factors to the spread of HEV. Participants highlighted that they are faced with a challenge of lack of toilets in the Havana informal settlement:

‘Most people there have a problem of not having toilets, they use plastics as their toilets and throw them away on the riverbeds special at nights and those practices can increase HEV spread in the community.’ (Participant 7, female, age 58 years)

Overcrowding in the informal settlement has a significant impact in the controlling of HEV. Participants expressed that the setting is overpopulated and that the available donated toilets are insufficient to meet the needs of the inhabitants:

‘A lot of us are sharing things like taps, toilets and we do not have enough toilets and water points.’ (Participant 3, male, age 33 years)

The accessibility to clean water and shared water points within the community play an important role in the management of communicable diseases. Participants stated that lack of adequate water within Havana informal settlement contributed to the spread of HEV as it limits people’s ability to perform hygienic practices:

‘Limited access to water compromises personal hygiene such as regular hand washing and food hygiene so people who don’t wash their hands, this will spread HEV in the community.’ (Participant 14, male, age 41 years)

**Community, social and cultural practices:** Participants stated that some community members are still not following the prescribed HEV prevention protocols. Moreover, the participants highlighted that even though community members are given correct health education, some cultural values and traditional beliefs compromise adherence to the HEV prevention protocols. This includes practices such as handshaking:

‘The community is given proper health education, however cultural practices and norms are the biggest challenge to control HEV example in our Oshiwambo tradition we believe that if a visitor comes at home, we have to shake their hands, and now after shaking hands they usually forget to wash their [hands] before handling food.’ (Participant 4, male, age 28 years)

Participants were concerned that some community members deliberately did not adhere to the HEV prevention measures in everyday life, for example, hygienic food handling practices and toilet hygiene and basic maintenance:

‘Despite the effort of health care workers to educate the public of HEV prevention measure such as food hygiene, some people are still leaving food open for a very long time which would expose it to flies which would land on food from unclean toilets which are sources of HEV infection.’ (Participant 12, male, age 48 years)

Most cultural practices involve daily community interactions, for example, traditionally in Oshiwambo, the whole family wash their hands in one bucket before eating. Elders wash their hands first before younger family members. Hence, this practice easily exposes the whole family to communicable diseases such as HEV:

‘People are still washing hand in one bucket before eating as family whereby the elders wash first and children are last, of which at the end of the day, it was likely for the whole family to acquire the HEV transmission.’ (Participant 4, male, age 28 years)

Participants indicated that social and cultural norms had a very important influence on the behaviour of the community. This includes the method of interaction in everyday life as a community. Participants further alluded that the community’s social norms and cultural practices regulate the community and public interactions at public spaces such as Bars and Cuca shops (Shebeens) where the community members meet for socialisation. It is culturally and socially accepted that Tombo liquor cups (traditional liquor) are shared, but there is no consistency in terms of hand washing and washing of shared Tombo cups:

‘In our community people usually drinks traditional liquor (Tombo) together where they normal share cups when drinking the liquor, but there are no proper handling hygienic practices which has a likelihood of transmitting the HEV.’ (Participant 12, male, age 48 years)

Other social and community practices that drive the spread of HEV are discrimination and stigma. Community leaders expressed that they have observed stigma against people with suspected and confirmed HEV by community members. Some community members stopped communicating with neighbours, distancing themselves from HEV suspects:

‘Hepatitis E came with a lot of challenges such as discrimination and victimisation of neighbours because of fear to get HEV from fellow neighbour and families.’ (Participant 1, male, age 45 years)

#### Theme 3: Action drivers for change

In the eradication of HEV, certain factors can facilitate or drive actions or behaviour. In this study, these action drivers included perceiving the benefits of acting and knowledge of HEV prevention methods. However, barriers that hinder potential action were also identified.

**Perceived benefits of taking action:** Community members learnt the benefits of taking action, which motivated them to adhere to HEV prevention protocols. Participants indicated that community members who understood the benefits of taking action such as the prevention of the transmission of HEV practised individual and collective self-care:

‘People started taking care of themselves; they started practicing general and personal hygiene to avoid community transmission. However, it has disrupted most of our lifestyles, people don’t visit quite as often not shaking hands, since it associated with HEV spread of the disease.’ (Participant 9, male, age 56 years)

Community leaders stated that individual participation in the prevention of HEV played a significant role and that it was a gradual process of increasingly adopting healthy behaviours. This includes personal hygiene practices such as handwashing, proper use of toilets, healthy eating and food hygiene:

‘As ways of HEV prevention, individuals started washing our hands with soap of course, consume food that contains more vitamins and clean water that the body needs and practicing environmental hygiene.’ (Participant 11, male, age 55 years)

**Perceived barriers to action:** Participants stated that community members have various challenges in fighting against HEV in the community. The discrimination towards the people suspected or confirmed with HEV was one of the barriers to successfully combating HEV community transmission. Stigma and discrimination were fuelled by myths and misconceptions. These misconceptions hindered the communication of accurate information:

‘Hepatitis E came with a lot of challenges such as discrimination, hating of neighbours because of fear to get HEV from fellow neighbour and families, however some stigma associated myths, which scientifically proven like some believed that through indirect contact like air born.’ (Participant 1, male, age 45 years)

Participants highlighted that a lack of understanding among the community members attributed to the spread of HEV in the community. Some participants believed that the information communicated was not effective in resulting in behaviour change:

‘Yes, at this point we need more effective information about the sanitation as some people do not understand the importance of toilet hygiene and environment hygiene.’ (Participant 8, female, age 52 years)

Community leaders highlighted that social class, poverty and unemployment were barriers to combating HEV. This is because the informal settlement has poor sanitation such as a lack of sufficient toilets. Some community members are also sharing toilets, making it difficult to maintain proper hygiene:

‘In the informal settlement most of residents are unemployed, and this economic status deprive the residents to afford to make their toilets and maintain proper hygiene.’ (Participant 8, female, age 52 years)

Participants highlighted the lack of resources in the community to combat the HEV including toilets, cleaning detergent or soaps and clean water:

‘It’s a bit difficult to control the virus because of lack of toilet and poor sewage drainage system in our environment, poor cleanness in the toilets no cleaning detergents and washing our hands soaps.’ (Participant 12, male, age 48 years)

Participants indicated that despite the effort of the MOHSS to educate the community, some community members are ignorant about adhering to HEV prevention protocols or resistant to change. Therefore, there is a need for continuous health education to the community members:

‘But not all the people do clean despite being educated on the importance of keeping toilets and persistent to use the toilet. We find ourselves sometimes the toilet is just dirty because the one that was supposed to clean the toilet is either not there or not willing to changes.’ (Participant 14, male, age 41 years)

**Knowledge of the prevention measures of hepatitis E virus:** Community leaders indicated that most of the community members have basic knowledge of the prevention of HEV community transmission. This includes the proper and adequate sanitation utilisation facilities such as toilets. Even though the community might be willing to utilise adequate sanitation, there are limited access to sanitation facilities, for example, sewage facilities:

‘Yes, the community is now aware of the disease, so we are trying as much as we can to make sure there’s a big change to lower the risk for contracting this disease, this included washing hands, use of toilets and personal hygiene.’ (Participant 3, male, age 33 years)

Participants eluded that continuous HEV community awareness remains crucial as there are some community members who are still struggling to change their behaviour towards the elimination of HEV. Participants felt that good practices should be reinforced:

‘First of all, maybe the Ministry of Health better to come and make awareness campaigns because some people are still not changing, for example still not cleaning their toilets, poor food hygiene and irregular hands washing with running water.’ (Participant 3, male, age 47 years)

The participants highlighted that the HEV epidemic brought a lot of changes in social and cultural norms and change of lifestyles. Community members had to change some of their unhygienic habits such as shaking of hands. However, this also had an impact on the social fabric of the community:

‘People started taking care of themselves; they started practising general and personal hygiene to avoid community transmission. The bad effect I would say it has disrupted most of our cultures and lifestyles as people don’t visit quite each other as usual due to HEV. Other changes were also brought in cultural practices, people don’t shake hands anymore since it will contribute to the spread of the disease.’ (Participant 1, male, age 55 years)

#### Theme 4: Actions

This theme involves the actions taken towards preventing the spread of HEV. The sub-themes include actions by individuals, community leaders and the community at large towards preventing the spread of HEV. Community members were the agents of change, whose collective actions can result in the elimination of the HEV in their community. Community leaders stated that there were some remarkable changes in the community to fight against HEV.

**Community actions:** The community actions were evident in the locations as they started keeping their donated toilets clean and prioritising the use of toilets. The community came up with an initiative to identify their needs as a team to combat HEV transmission including requesting donations. Participants further expressed they received different donations from both private and government funds:

‘As community members we received donations from the government for toilets and the municipality also brought water containers next to our houses; they also brought taps with temporary water.’ (Participant 6, female, age 49 years)

Behavioural change in the community remained crucial in the fight against the epidemic. Participants stated that in many cases, there were significant changes in the community’s behaviour towards healthy lifestyles such as proper handwashing and environmental hygiene:

‘Most importantly started regular washing hands with soap, cleaning the toilet after using it, keeping our environment clean and making use of bins. The community are really trying this and practicing safe general hygiene and this will prove effective way of managing the epidemic.’ (Participant 11, male, age 55 years)

The community’s collective actions yielded better results in the fight against the HEV and maintained the donated items such as toilets and practising hand washing.

**Community leader actions:** Community leaders were advocates in fighting against HEV; they were involved in conducting public education through community meetings. Community leaders saw themselves as agents for health as they were trained in the prevention of HEV:

‘We have been educating the community by raising awareness, educating the community on importance of cleaning toilets after use of toilet, promoting environmental hygiene; enforcing the of use soap and other chemicals that you can use to clean the toilet.’ (Participant 5, male, age 38 years)

**Individual actions:** Participants indicated that individual business owners and household members adopted healthy practices and lifestyles that were aimed at mitigating the HEV community transmission:

‘Majority of us started washing our hands with soap of course, consuming hygienic well-prepared food and drinking cleans water and practicing environmental hygiene such as regular cleaning toilets.’ (Participant 11, male, age 55 years)

‘Some businesses man they started selling traditional liquor individually which was a bit challenging. People started limiting the continuous families and neighbour’s visit, some even they hesitated offering food to the victors specially the one that easily transmit the HEV.’ (Participant 6, male, age 49 years)

**Public health and governmental actions:** The participants appreciated the government and municipal contribution in supplying the community with sanitation facilities such as adequate toilets, although this was not sufficient:

‘People were given toilets by the Windhoek Municipality; two toilets are to be shared by 20 houses and even though they were given toilets some people are still not using them due to overcrowding.’ (Participant 6, male, age 49 years)

Community leaders stated that the MOHSS has been conducting community HEV awareness campaigns and meetings in the community. The meetings were more focused on sensitising the community rather than prevention measures:

‘Yes, people had been given health education by MOHSS or health care workers and after community awareness most community members, started washing hands, using toilet. People also changed our harmful cultural practices such shaking hands and washing hands together.’ (Participant 9, male, age 26 years)

The participants highlighted that the government provided additional toilets and clean water to the community as remedial actions to curb HEV community transmission:

‘The toilets were donated to us by the municipality that were allocated 20 houses per toilet and government also gave us extra water tank during the HEV epidemic to increase access to safe drinking water.’ (Participant 9, male, age 26 years)

## Discussion

The study objective was to investigate community leaders’ experiences with the spread of the HEV in the Havana informal settlement, Khomas region, Namibia, using the HBM as an underpinning framework. As important participants in HEV prevention initiatives, community leaders considered the overall level of risk knowledge in the community, the environmental elements that contribute to the spread of HEVs and the measures implemented to avoid the disease.

One of the study’s key themes was risk awareness, which is the understanding of possible dangers that could cause injury. The participants’ comprehension of HEV’s symptoms, indications and modes of transmission was remarkable; they identified inadequate water supply, poor sanitation and subpar environmental hygiene as key causes of the outbreak. This awareness is consistent with the HBM, which holds that when people believe their health is in danger, they are more likely to take actions that would improve it.^25^ However, some community members exhibited limited knowledge, with misconceptions about the disease, such as associating coughing with HEV. Previous literature has noted that a lack of accurate information can hinder effective prevention efforts, as individuals may not recognise the importance of hygiene practices in mitigating the spread of the virus.^26^

In addition, the study highlighted the importance of knowledge as a critical factor in community leaders’ ability to educate others about HEV. This corresponds with past studies, which highlighted that knowledge is defined as what is known by an individual that applies to facts or ideas acquired through study, investigation, observation or experience.^26^ Community leaders need to have sufficient knowledge about HEV because they serve as focal points for information dissemination in their communities.^27^ The study found that while many participants were aware of the disease’s transmission routes, some community leaders and members lacked adequate knowledge about the signs and symptoms of HEV. This finding aligns with an earlier study, which highlighted that gaps in understanding transmission routes can contribute to misinformation within the community, thereby complicating prevention efforts.^28^

Contextual factors, including environmental conditions and socio-cultural practices, were identified as critical drivers of HEV transmission. The study revealed that traditional norms, such as communal handwashing and sharing drinking vessels, pose risks for cross-infection. These practices are deeply rooted in the community’s culture, making it challenging to implement preventive measures. This aligns with previous literature, which emphasised that the custom of sharing a handwashing bucket before meals, while culturally significant, can facilitate the transmission of HEV.^29^ This highlights the need for culturally sensitive health education that respects local practices while promoting safer alternatives.

Moreover, the study indicated that socio-economic conditions significantly influence the spread of HEV. Participants noted that overcrowding, lack of sanitation facilities and limited access to clean water were prominent contributing factors fuelling the outbreak. Research has shown that structural determinants and socio-economic conditions can drive communicable diseases.^30^ Individuals with lower socio-economic status, particularly those residing in overcrowded informal areas, face increased susceptibility to HEV because of their living conditions.^10^

Action drivers for change emphasised the perceived benefits of preventive measures, with community members initiating practices like regular handwashing and improved food hygiene. Participants expressed a clear understanding of the significance of taking proactive measures against HEV, recognising that these actions could prevent both individual and community transmission. This understanding is crucial, as the HBM emphasises that individuals are more likely to adopt health-promoting behaviours when they perceive the benefits of such actions to outweigh the barriers. However, challenges such as discrimination against those suspected of having HEV and a lack of resources, including toilets and clean water, hinder effective prevention. Discrimination and stigma surrounding HEV can deter individuals from seeking help or disclosing their status, further complicating efforts to control the outbreak. The study found that community leaders identified discrimination as a significant obstacle to combating HEV transmission. This echoes previous findings from other studies, which have shown that stigma can lead to mental distress and social isolation for affected individuals.^30,31^

The lack of resources emerged as a primary contributor to the prevalence of HEV in the Havana informal settlement. Despite efforts to distribute toilets to low-income earners, there remains an inadequacy in the provision of these essential facilities for all community members.^32^ Many residents resort to using makeshift facilities for defecation, exacerbating the risk of HEV transmission. Current results align with previous research, which demonstrated the clear link between poverty and the spread of HEV, as the majority of individuals in informal settlements are either unemployed or low-income earners.^16^ Another study conducted in 2021 found that economic disadvantage renders the community particularly susceptible to HEV because of living conditions characterised by poor hygiene and limited access to sanitation facilities.^32^

Furthermore, the findings emphasise the importance of targeted health education to address knowledge gaps and foster community engagement in HEV prevention. Community leaders play a crucial role in disseminating information and mobilising resources, yet their efforts can be undermined by misinformation and cultural practices that conflict with health recommendations. According to earlier research, preventative efforts are hampered by cultural customs including communal handwashing and misinformation.^25,33^ The study revealed that while community-driven actions and government support were evident, the ongoing lack of access to essential sanitation facilities in informal settlements necessitates continued efforts to bridge these gaps. In the same vein, other studies have emphasised the role of infrastructure in disease prevention,^31^ highlighting the need for improved sanitation to reduce health risks.^34,35^

Research should focus on local knowledge, attitudes and behaviours related to HEV transmission and prevention. Community engagement is crucial, as it fosters collaboration between healthcare providers and residents, empowering individuals to take ownership of their health.^36^ For healthcare providers, this means advocating for health education and building trust, overall, leading to improved health outcomes and a more informed community.

### Study strengths and limitations of the study

The study’s strengths include its qualitative methodology and targeted context, which provide in-depth perspectives on HEV prevention from local leaders. However, its limitations include potentially limited generalisability of the findings to broader populations beyond the Havana informal settlement.

## Conclusion

This study’s result highlights the necessity of a thorough strategy for HEV prevention that raises community awareness and expands access to sanitary facilities. Effective measures to lower HEV transmission can be put into place by encouraging cooperation and understanding among stakeholders. These initiatives are essential for empowering people and guaranteeing the welfare of those who live in the Havana informal settlement. Long-term health promotion requires enduring collaborations between organisations, the government and the community. Additionally, these findings may have broader implications for the prevention of other communicable diseases.
